# Delay-Compensated Lane-Coordinate Vehicle State Estimation Using Low-Cost Sensors

**DOI:** 10.3390/s25196251

**Published:** 2025-10-09

**Authors:** Minsu Kim, Weonmo Kang, Changsun Ahn

**Affiliations:** School of Mechanical Engineering, Pusan National University, Busan 46241, Republic of Korea; kmsms5051@pusan.ac.kr (M.K.); wmkang@pusan.ac.kr (W.K.)

**Keywords:** lane coordinate, extended kalman filter, delay compensator, sensor fusion

## Abstract

Accurate vehicle state estimation in a lane coordinate system is essential for safe and reliable operation of Advanced Driver Assistance Systems (ADASs) and autonomous driving. However, achieving robust lane-based state estimation using only low-cost sensors, such as a camera, an IMU, and a steering angle sensor, remains challenging due to the complexity of vehicle dynamics and the inherent signal delays in vision systems. This paper presents a lane-coordinate-based vehicle state estimator that addresses these challenges by combining a vehicle dynamics-based bicycle model with an Extended Kalman Filter (EKF) and a signal delay compensation algorithm. The estimator performs real-time estimation of lateral position, lateral velocity, and heading angle, including the unmeasurable lateral velocity about the lane, by predicting the vehicle’s state evolution during camera processing delays. A computationally efficient camera processing pipeline, incorporating lane segmentation via a pre-trained network and lane-based state extraction, is implemented to support practical applications. Validation using real vehicle driving data on straight and curved roads demonstrates that the proposed estimator provides continuous, high-accuracy, and delay-compensated lane-coordinate-based vehicle states. Compared to conventional camera-only methods and estimators without delay compensation, the proposed approach significantly reduces estimation errors and phase lag, enabling the reliable and real-time acquisition of vehicle-state information critical for ADAS and autonomous driving applications.

## 1. Introduction

Advancements in Advanced Driver Assistance Systems (ADASs) and fully autonomous driving (AD) are critical for safe and efficient future transportation systems [1,2]. Functions such as Lane Keeping Assist (LKA), Adaptive Cruise Control (ACC), and urban autonomous driving rely heavily on accurate and robust perception of the vehicle’s state within its environment [3]. For stable operation, it is essential to estimate not only the vehicle’s absolute position in a global coordinate system but also its relative state with respect to the lane. In particular, lateral position, lateral velocity, and heading angle in a lane coordinate system (curvilinear coordinates) provide more intuitive and useful information for path planning and control algorithms [4,5,6]. Among these, lateral velocity relative to the lane is a critical state for motion prediction but cannot be directly measured with typical onboard sensors.

Current state-of-the-art approaches achieve centimeter-level accuracy by fusing high-cost sensors such as HD maps, LiDAR, radar, and GPS [2,7,8,9]. For example, Bersani et al. proposed a framework that integrates pre-built road maps with multi-sensor data to estimate the vehicle and surrounding obstacles in the lane coordinate system [4]. However, these methods are costly and lack scalability for general-purpose vehicles. Consequently, there is an urgent need for map-less state estimation methods that utilize only low-cost sensors, such as cameras and IMUs [10].

Low-cost sensor-based approaches face two main challenges. First, many existing methods rely on simple kinematic models, such as the Constant Turn Rate and Acceleration (CTRA) model [11,12,13]. While sufficient for stable driving, these models fail to capture real vehicle behavior during sudden steering or evasive maneuvers, where vehicle dynamics—including tire lateral forces and slip—dominate. This limitation can severely degrade estimation performance. Recent studies emphasize the importance of dynamic models that accurately reflect a vehicle’s physical characteristics for precise estimation, particularly of lateral velocity [4,9,14,15].

The second challenge is the inherent delay of vision-based sensors. Cameras and other perception sensors have slow and variable data acquisition rates due to the large amount of data processing, leading to unevenly delayed measurements [16,17]. Such delays can degrade perception performance and destabilize control loops [18]. Various attempts have been made to solve the problems caused by such delays. For example, to fundamentally reduce the delay itself, a wide range of methods have been explored [19,20], from traditional computer vision techniques for fast lane detection to modern approaches that use deep learning with lightweight models [21] or fusing different feature extraction methods [22]. However, even with these efforts to reduce processing time, a slight time delay can still lead to a significant performance degradation in vehicle control systems.

Another approach involves compensating for signal delays that are unavoidable due to heavy computational loads or communication processes. Currently, a widely used method for signal delay compensation is to modify the timestamp of the input signal by the amount of the delay [23,24,25]. However, most research on vehicle state estimation using signal delay compensation has not yet incorporated the vehicle’s dynamic characteristics [26,27]. Although there was a study by Wang et al. that considered vehicle dynamics and vision sensor signal delay to estimate the in-lane lateral position and angle [16], it did not estimate the lane-based lateral velocity, which still leaves a limitation in fully explaining the vehicle’s in-lane behavior.

**Figure 1:**
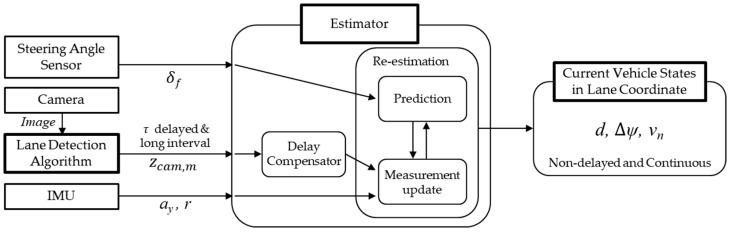
Estimator structure.

To address these challenges, this study makes a significant contribution by proposing a novel lane-coordinate-based state estimator that operates using only low-cost sensors (a camera, IMU, and steering angle sensor) without relying on HD maps. The core of our approach is the tight integration of a vehicle dynamics-based bicycle model within an Extended Kalman Filter (EKF). This foundation overcomes the limitations of traditional kinematic models, and its most critical achievement is the accurate, real-time estimation of the vehicle’s lateral velocity relative to the lane—a key state for motion prediction that cannot be directly measured with typical onboard sensors. By robustly estimating this crucial, unmeasurable state alongside lateral position and heading angle, our method provides a comprehensive understanding of the vehicle’s dynamic behavior within the lane, which is essential for advanced vehicle control. Furthermore, by using the dynamics model to propagate the state forward during sensor processing periods, the estimator effectively compensates for the signal delay from the vision system, ensuring the final output remains timely and accurate. To support practical implementation, a computationally efficient camera processing pipeline is also introduced, including lane segmentation using a pre-trained network and lane-based state extraction. The overall structure of the proposed estimator is illustrated in Figure 1, which highlights the integration of signals from low-cost sensors, the proposed estimator contains adaptive delay compensation and an asynchronous compensation structure, and estimation results include lateral velocity about the lane.

The proposed estimator provides continuous, accurate, and delay-free lane-coordinate-based vehicle states, including the unmeasurable lateral velocity, which are directly applicable to ADAS and autonomous driving. Validation with real vehicle data on straight and curved roads demonstrates the estimator’s effectiveness under realistic driving conditions and confirms its real-time performance.

## 2. Vehicle Model for Estimator

### 2.1. Road-Following Vehicle Model in Vehicle Coordinates

A reliable vehicle model is essential for state estimation. A commonly used representation is the road-following vehicle model, which assumes small variations in lateral deviations and heading angle, ensuring the validity of linear approximations [28,29]. This model describes the lateral behavior of a vehicle based on a given steering angle. It is particularly suitable for explaining a vehicle’s lane-based state while considering its dynamic characteristics, as it defines the state in terms of lateral error relative to a given path or lane. The model is expressed as:

(1) $$\left[\begin{array}{c} \dot{d}\\ \dot{v}\\ \dot{\psi }-{\dot{\psi }}_{d}\\ \dot{r} \end{array}\right]=\left[\begin{array}{cccc} 0 & 1 & u & 0\\ 0 & \frac{-(C_{\alpha f}+C_{\alpha r})}{mu} & 0 & \frac{-aC_{\alpha f}+bC_{\alpha r}}{mu}-u\\ 0 & 0 & 0 & 1\\ 0 & \frac{-aC_{\alpha f}+bC_{\alpha r}}{I_{z}u} & 0 & \frac{-(a^{2}C_{\alpha f}+b^{2}C_{\alpha r})}{I_{z}u} \end{array}\right]\left[\begin{array}{c} d\\ v\\ \psi -\psi_{d}\\ r \end{array}\right]+\left[\begin{array}{c} 0\\ \frac{C_{\alpha f}}{m}\\ 0\\ \frac{aC_{\alpha f}}{I_{z}} \end{array}\right]\left[\delta_{f}\right]+\left[\begin{array}{c} 0\\ 0\\ -1\\ 0 \end{array}\right]\left[r_{d}\right]$$

where *d* is lateral displacement relative to the lane centerline, *v* is the lateral velocity, *r* is the yaw rate, *ψ* is the vehicle heading angle, *ψ_d_* is the desired heading angle, *u* is the longitudinal velocity, *C_αf_* and *C_αr_* are the front and rear tire cornering stiffness, *m* is the vehicle mass, *a* and *b* are the distances from the front and rear axles to the center of gravity(CG), *I_z_* is the yaw moment of inertia, *δ_f_* is the front steering angle, *r_d_* is the desired yaw rate along the lane center, and the longitudinal velocity *u* is assumed constant. These variables are also summarized in Table 1.

This formulation is expressed in the vehicle coordinate system and will serve as the basis for its representation in the lane coordinate system, described next.

### 2.2. Vehicle Model in Lane Coordinates

**Figure 2:**
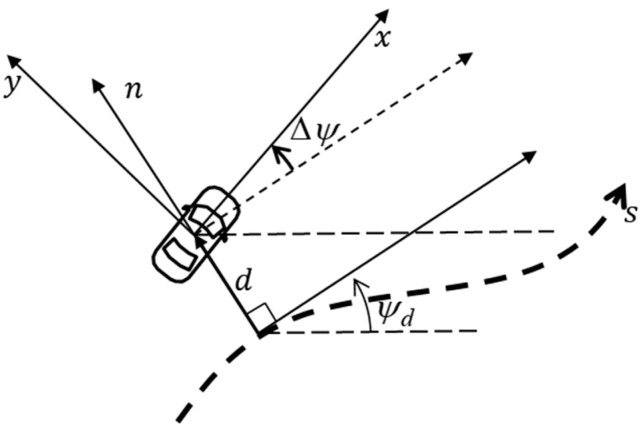
Vehicle expressed in lane coordinate.

To represent the vehicle model in the lane coordinate system, as shown in Figure 2, additional states must be introduced: the lateral displacement from the lane center and the corresponding lateral velocity. These are expressed as:

(2) $$\dot{d}=v_{n},$$

(3) $$v_{n}=u\sin(\Delta \psi )+v\cos(\Delta \psi ).$$

where Δ*ψ* = *ψ − ψ_d_* is the heading angle offset between the vehicle and the lane center. Assuming constant longitudinal velocity, the derivative of the vehicle’s lateral velocity relative to the lane center is:

(4) $${\dot{v}}_{n}=\dot{v}\cos(\Delta \psi )+u\cos(\Delta \psi )\Delta \dot{\psi }-v\sin(\Delta \psi )\Delta \dot{\psi }.$$

For small Δ*ψ*, Equation (4) can be simplified using the relation from (1).

(5) $${\dot{v}}_{n}=-\frac{C_{\alpha f}+C_{\alpha r}}{mu}v+\frac{bC_{\alpha f}-aC_{\alpha r}}{mu}r+\frac{C_{af}}{m}\delta_{f}-\Delta \psi v(r-r_{d})-ur_{d},$$

Because the lane curvature *κ* changes gradually, it is assumed constant over short intervals, yielding the relation between desired yaw rate, curvature, and longitudinal velocity:

(6) $$r_{d}=u\kappa ,\;\dot{\kappa }\approx 0.$$

Substituting into (5) gives:

(7) $${\dot{v}}_{n}=-\frac{C_{\alpha f}+C_{\alpha r}}{mu}v+\frac{bC_{\alpha f}-aC_{\alpha r}}{mu}r+\frac{C_{af}}{m}\delta_{f}-\Delta \psi v(r-u\kappa )-u^{2}\kappa .$$

The heading angle offset relative to the lane center is expressed as:

(8) $$\Delta \dot{\psi }=\dot{\psi }-{\dot{\psi }}_{d}=r-r_{d}=r-u\kappa ,$$

Combining the above, the nonlinear vehicle model in lane coordinates is expressed as:

(9) $$\dot{X}=f(X,U)=\left[\begin{array}{l} v_{n}\\ r-u\kappa \\ -\frac{C_{\alpha f}+C_{\alpha r}}{mu}v+\frac{bC_{\alpha r}-aC_{\alpha f}}{mu}r-\Delta \psi vr+\frac{C_{af}}{m}\delta_{f}+\Delta \psi vr_{d}-u^{2}\kappa \\ -\frac{C_{\alpha f}+C_{\alpha r}}{mu}v+(\frac{bC_{\alpha r}-aC_{\alpha f}}{mu}-u)r+\frac{C_{af}}{m}\delta_{f}\\ \frac{bC_{\alpha r}-aC_{\alpha f}}{I_{z}u}v-\frac{a^{2}C_{\alpha f}+b^{2}C_{\alpha r}}{I_{z}u}r+\frac{aC_{af}}{Iz}\delta_{f}\\ 0 \end{array}\right]$$

$$where\;X={\left[\begin{array}{cccc} d & \Delta \psi & v_{n} & \begin{array}{ccc} v & r & \kappa \end{array} \end{array}\right]}^{T},\;U=\delta_{f}.$$

Therefore, in addition to *v* and *r*, curvature *κ* must also be included as a state. While *κ* can be directly extracted from camera images, such measurements suffer from delays and low update rates, making them unsuitable as direct model inputs. Moreover, assuming a zero-curvature rate of change prevents accurate estimation using the system model alone. Therefore, curvature is incorporated through a measurement model, with its implications addressed in the estimator design section.

The derivation above establishes a vehicle model expressed in lane coordinates, where lateral dynamics and road curvature are explicitly incorporated. Since curvature cannot be accurately predicted from the system model alone, reliable lane information is required. The following section introduces the lane detection framework, which provides the curvature and heading references necessary for the estimator design.

## 3. Vehicle State Estimator in Lane Coordinates

### 3.1. Lane Detection Algorithm

To provide the lane information required for curvature and heading references in the estimator, we employ a camera-based detection method. Unlike other sensors, a camera does not directly measure lane geometry; instead, image data must be processed to extract relevant lane features. Since the system operates on a moving vehicle, real-time performance is critical, and, therefore, the computational efficiency of the detection algorithm is a key concern.

**Figure 3:**
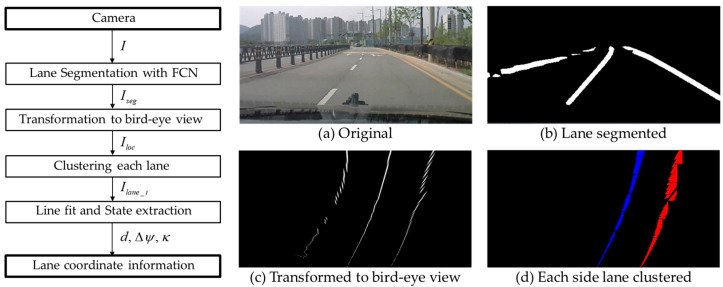
Overview of the lane detection algorithm pipeline. The process consists of three main stages: (1) lane segmentation using a pre-trained deep neural network, (2) transformation of the segmented image into the vehicle’s local coordinate system, and (3) lane shape extraction via point clustering and polynomial fitting. The resulting polynomial representation provides the lane geometry—including lateral offset, heading angle deviation, and curvature—expressed in the vehicle coordinate system.

We employ a method that balances robustness and efficiency, comprising three main steps: lane segmentation, coordinate transformation, and lane shape extraction. The overall procedure is illustrated in Figure 3.

First, lane segmentation is performed using a pre-trained Fully Convolutional Network (FCN) with ResNet50 as the backbone. This network is a highly accessible model commonly used for image classification and segmentation [19,21]. Given these characteristics, we adopted this segmentation-based methodology as a representative example of a lane detection method that introduces signal delay. Specifically, we chose FCN as the base architecture and used a pre-trained ResNet50 provided by PyTorch v1.12 as the backbone. This model was then fine-tuned on a lane recognition dataset [30,31] and subsequently utilized as a lane segmentation network. The network is trained to segment lane components from input road images, and the output retains only the pixels corresponding to lane markings:

(10) $$I_{seg}=net(I).$$

Because the segmented image is expressed in a distorted camera coordinate system, it is transformed into the vehicle’s local coordinate system. Using *info.*, the camera’s installation parameters, orientation, and distortion characteristics, the image portion between the vanishing point and the vehicle’s position is projected onto the horizontal plane:

(11) $$I_{loc}=transformation(I_{seg},\;info.).$$

Even after projection, lanes may not be distinctly separated. An adjacent-point searching and clustering method is therefore applied to distinguish the left and right lanes:

(12) $$I_{lane\_i}=clustering(I_{loc})\;\;(i=left,\;right).$$

Finally, a third-order polynomial is fitted to the lane shape. With an appropriate meter-to-pixel ratio, the lane geometry is obtained in the vehicle’s local coordinate system:

(13) $$P_{i}=r_{p2m}linefit(I_{lane\_i}),\;i=\{left,\;right\}.$$

This polynomial representation directly yields the required lane-based states: lateral offset *d*, heading angle Δ*ψ*, and curvature *κ*. Under the small-angle assumption for Δ*ψ*, these states are computed as:

(14) $$d_{i}=P_{i}(0),$$

(15) $$\Delta \psi_{i}=\arctan(P_{i}\prime (0))\approx P_{i}\prime (0),$$

(16) $$\kappa_{i}=P_{i}\prime \prime (0)/{\left(1+P_{i}\prime {\left(0\right)}^{2}\right)}^{3/2}\approx P_{i}\prime \prime (0),$$

$$where\;P_{i}(x)=p_{3\_i}x^{3}+p_{2\_i}x^{2}+p_{1\_i}x+p_{0\_i}\;\;(i=left,\;right),\;$$

$$\Delta \psi \approx 0,\;P_{i}\prime {\left(0\right)}^{2}\approx 0.$$

Alternatively, they can be expressed in terms of the polynomial coefficients, with improved accuracy obtained by using the midpoint between the left and right lanes:

(17) $$d=(p_{0\_left}+p_{0\_right})/2,$$

(18) $$\Delta \psi =(p_{1\_left}+p_{1\_right})/2,$$

(19) $$\kappa =(2p_{2\_left}+2p_{2\_right})/2.$$

In summary, the algorithm provides accurate lane-based states, but the processing introduces non-negligible delays due to its computational complexity. These delays lead to errors in estimating the vehicle’s instantaneous state relative to the lane. Therefore, it is necessary to design an estimator that can accurately estimate the current state by properly accounting for this delay. For accurate estimation, the exact amount of delay must be considered. To precisely measure this delay, we included a function to measure the image processing time when implementing the algorithm described above. The following section presents a methodology to compensate for this delay through estimator design.

### 3.2. Vehicle State Estimator

To achieve robust estimation in the presence of sensor delays and signal faults, careful estimator design is required. As derived in Equation (9), the vehicle states of interest are governed by nonlinear dynamics. At the same time, real-time performance is essential for vehicle applications, necessitating an estimation framework that balances accuracy with computational efficiency.

In this study, the Extended Kalman Filter (EKF) is adopted. The EKF is particularly well suited to this problem because it can accommodate nonlinear system models while maintaining relatively low computational cost, avoiding the need for sigma-point propagation as in the Unscented Kalman Filter [32]. The EKF operates through prediction and correction steps based on local linearization of the nonlinear model.

The state vector *x* is defined to include the target lane-coordinate-based states and their auxiliary dynamics, rearranged from the relationships derived in Section 2:

(20) $$\dot{X}=f(X,U)=\left[\begin{array}{c} x_{3}\\ x_{5}-ux_{6}\\ -\frac{C_{\alpha f}+C_{\alpha r}}{mu}x_{4}+\frac{bC_{\alpha r}-aC_{\alpha f}}{mu}x_{5}-x_{2}x_{4}x_{5}+\frac{C_{af}}{m}\delta_{f}+ux_{2}x_{4}x_{6}-u^{2}x_{6}\\ -\frac{C_{\alpha f}+C_{\alpha r}}{mu}x_{4}+(\frac{bC_{\alpha r}-aC_{\alpha f}}{mu}-u)x_{5}+\frac{C_{af}}{m}\delta_{f}\\ \frac{bC_{\alpha r}-aC_{\alpha f}}{I_{z}u}x_{4}-\frac{a^{2}C_{\alpha f}+b^{2}C_{\alpha r}}{I_{z}u}x_{5}+\frac{aC_{af}}{Iz}\delta_{f}\\ 0 \end{array}\right]$$

$$where\;X={\left[\begin{array}{cccccc} x_{1} & x_{2} & x_{3} & x_{4} & x_{5} & x_{6} \end{array}\right]}^{T}={\left[\begin{array}{cccc} d & \Delta \psi & v_{n} & \begin{array}{ccc} v & r & \kappa \end{array} \end{array}\right]}^{T},\;U=\delta_{f}.$$

The measurable outputs *Z*, obtained from the IMU and camera sensors, are related to the state vector as:

(21) $$Z=h(X,U)=\left[\begin{array}{c} -\frac{C_{\alpha f}+C_{\alpha r}}{mu}x_{4}+\frac{bC_{\alpha r}-aC_{\alpha f}}{mu}x_{5}+\frac{C_{af}}{m}\delta_{f}\\ x_{5}\\ x_{1}\\ x_{2}\\ x_{6} \end{array}\right]$$

$$where\;Z={\left[\begin{array}{cc} z_{IMU} & z_{cam} \end{array}\right]}^{T},\;z_{IMU}=\left[\begin{array}{cc} a_{y} & r \end{array}\right],\;z_{cam}=\left[\begin{array}{ccc} d & \Delta \psi & \kappa \end{array}\right],\;a_{y}=\dot{v}+ur$$

By introducing the process noise covariance *Q* and measurement noise covariance *R*, with respective noise terms *w_k_* and *v_k_*, the EKF problem formulation can be expressed as:

(22) $$\left\{\begin{array}{l} X_{k}=f(X_{k-1},U_{k})+w_{k},\\ Z_{k}=h(X_{k},U_{k})+v_{k}. \end{array}\right.$$

Accordingly, the EKF proceeds with prediction and measurement update steps [33]. First, during the prediction step, the nonlinear system is linearized for state propagation:

(23) $$F_{k}={\left.\frac{\partial f}{\partial x}\right|}_{{\hat{X}}_{k-1|k-1},U_{k}},\;H_{k}={\left.\frac{\partial h}{\partial x}\right|}_{{\hat{X}}_{k|k-1},U_{k}}.$$

The predicted state and covariance are then updated as:

(24) $$\left\{\begin{array}{l} {\hat{X}}_{k|k-1}=f({\hat{X}}_{k-1|k-1},U_{k}),\\ {\hat{P}}_{k|k-1}=F_{k}P_{k-1|k-1}{F_{k}}^{T}+Q_{k}. \end{array}\right.$$

Next, during the measurement update step, the state and covariance are corrected using the measurement information:

(25) $$\left\{\begin{array}{l} {\hat{X}}_{k|k}={\hat{X}}_{k|k-1}+K_{k}(Z_{k}-h({\hat{X}}_{k|k-1})),\\ P_{k|k}=(I-K_{k}H_{k}){\hat{P}}_{k|k-1}, \end{array}\right.$$

$$where\;K_{k}={\hat{P}}_{k|k-1}{H_{k}}^{T}S_{k}^{-1},\;S_{k}=H_{k}{\hat{P}}_{k|k-1}{H_{k}}^{T}+R_{k}.$$

A key challenge arises from the fact that the sensors operate at different update rates. In particular, camera-based measurements incur a significant delay due to image processing. To overcome these issues, the proposed framework incorporates two dedicated strategies, which will be described in the following subsection.

### 3.3. Compensation for Asynchronous Measurements

In conventional approaches, when a sensor such as a camera operates at a slower update frequency than other sensors, two options are typically used: (1) generating virtual measurements through zero-order hold or extrapolation, or (2) forcing the entire estimator to operate at the slower frequency. Both approaches have notable drawbacks. Virtual measurements may accumulate errors over time, while slowing the estimator prevents effective use of higher-frequency sensor information and may hinder downstream applications, such as control systems, that require fast updates.

To overcome this limitation, the proposed methodology omits the measurement update whenever a new camera measurement is unavailable and performs only the model-based prediction step of the EKF, as expressed in Equation (26):

(26) $$\begin{array}{l} {\hat{X}}_{k|k}={\hat{X}}_{k|k-1},\\ P_{k|k}={\hat{P}}_{k|k-1} \end{array}$$

This approach eliminates the errors introduced by virtual measurements while still enabling continuous estimation of vehicle states using high-frequency inputs from the IMU and steering angle sensors.

Naturally, the absence of measurements introduces concerns about observability. However, since this study assumes that no signal loss persists beyond a single measurement period, periodic observability is sufficient for reliable estimation. Observability was verified by conducting a rank test on the observability matrix *O*, defined using the linearized system matrix *F* and measurement matrix *H*, as given in Equation (27):

(27) $$O={\left[\begin{array}{cccc} H & HF & \ldots & HF^{n-1} \end{array}\right]}^{T}$$

### 3.4. Compensation for Delayed Measurements

As discussed earlier, the processing of camera images to extract lane information introduces an inherent signal delay. This delay, combined with errors caused by the limited field of view, can significantly reduce the accuracy of vehicle state estimation relative to the lane. Accordingly, proper delay compensation is essential.

In the proposed framework, delay compensation is achieved by correcting the timestamp of the delayed measurement and performing a re-estimation of the state from the corrected timestamp to the current. Specifically, if the processing delay of the camera is denoted as *τ*, the corrected measurement timestamp is given by Equation (28):

(28) $$z_{cam,true}(t-\tau )=z_{cam,m}(t)$$

In this study, only the processing delay of the camera is considered, as transmission delays are negligible. Since the image processing time required to extract lane information is known, the exact delay *τ* can be determined for each incoming measurement. By correcting the timestamp, the lane measurement can be aligned with the actual time at which it was captured.

**Figure 4:**
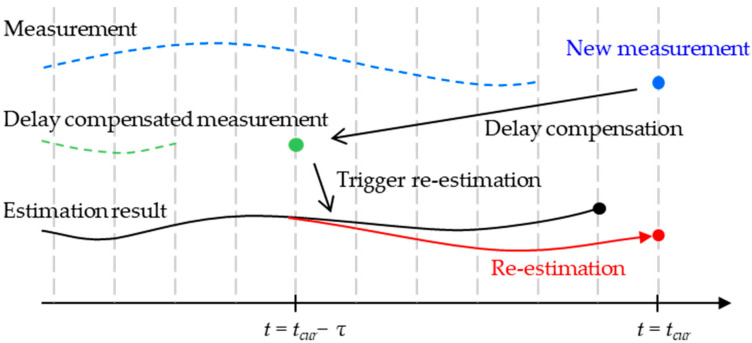
Conceptual illustration of the delay compensation process.

To incorporate this corrected measurement into the current state estimate, the system performs a re-estimation over the interval *t* = [*t_cur_* − *τ*, *t_cur_*], as illustrated in Figure 4 and expressed in Equation (29):

(29) $$\hat{X}(t+\Delta t)=EKF(X(t),\;U(t),\;Z(t),\;P(t))$$

During this re-estimation process, measurement updates are applied whenever new measurements are available. However, since only the camera signal is delay-compensated in this study, the procedure consists primarily of time updates using steering angle and IMU measurements, except for the initial step that incorporates the delay-corrected camera measurement.

## 4. Validation

### 4.1. Experiment Configuration

The proposed estimator was validated using data collected in real-world driving environments. This approach ensures that robustness is evaluated against actual signal faults encountered in practice, rather than against artificially generated signal errors.

**Figure 5:**
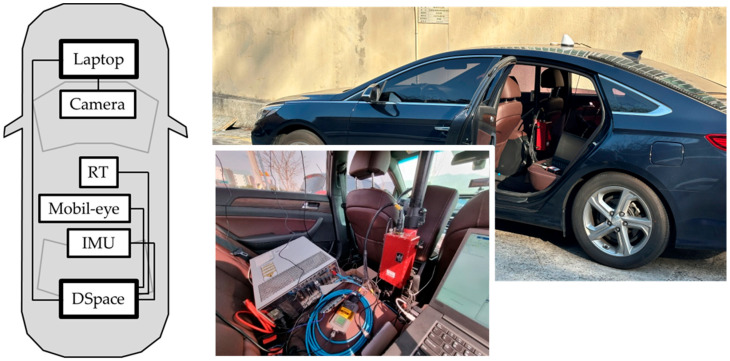
Experimental setup for data collection and validation.

For the experimental setup, a medium-sized sedan was equipped with the necessary sensors and devices. An OxTS RTK-GNSS (RT-K) system and a high-definition (HD) lane map were used to obtain ground-truth vehicle states, as shown in Figure 5. All validation data—including RT-K, IMU, and steering angle sensor signals—were logged via a dSPACE AutoBox using high-speed CAN. The AutoBox is a platform developed to support real-time Hardware-in-the-Loop (HIL) testing for vehicle Electronic Control Units (ECUs). Signals input into this device are recorded along with the device’s internal time, allowing the recorded data to be processed based on the precise time of input. Furthermore, in this study, it was assumed that the time taken for signals from each sensor, including the camera, to be transmitted via wired communication is very small and can therefore be considered negligible. This means that the estimator can process all sensor signals based on their generation time. The only signal delay that needs to be considered in this research is the processing delay that occurs during the extraction of the vehicle’s lane-based state from the camera image. The camera was connected to a laptop interfaced with the AutoBox, ensuring that all measurements were synchronized. The RT-K system provides highly accurate position and state information, which, when combined with the HD lane map, enables precise determination of the vehicle’s true states in the lane coordinate system. In addition, the estimator implementation required vehicle parameters (e.g., mass, inertia, tire stiffness). For this, vehicle parameters identified from prior dynamic behavior test data were used, as presented in Table 2.

Validation experiments were conducted on public roads featuring both straight and curved sections. To ensure sufficiently rich excitation of lateral dynamics, the vehicle was driven with maneuvers such as sine-wave steering within the lane and a Double-Lane Change (DLC), such as overtaking a preceding vehicle. Driving speeds ranged between 30 and 60 kph. Consequently, each scenario was composed of driving data lasting between 10 and 18 s and a trajectory length of 100 to 250 m. The data for each scenario was of sufficient length to fully exhibit the characteristics of the corresponding maneuver, making it suitable for providing meaningful validation results for lateral velocity estimation.

Because this study introduces a new lane-based vehicle state estimator with delay compensation, the vehicle states directly extracted from the camera (without estimation) were used as the first baseline for comparison. However, the lateral velocity *v_n_* in the lane coordinate system cannot be directly measured by any onboard sensor. Therefore, it was assumed that the vehicle’s lateral slip velocity *v* was sufficiently small to be neglected. Under this assumption, the baseline *v_n_* was computed using the vehicle’s longitudinal velocity *u* and the camera-measured heading angle deviation Δ*ψ*, as shown in Equation (30):

(30) $$Cam\;only=\left[\begin{array}{c} d\\ \Delta \psi \\ u\sin(\Delta \psi ) \end{array}\right]$$

Additionally, to evaluate the effect of the proposed delay compensation, estimation results obtained without applying delay compensation were used as a second baseline. This allowed direct assessment of the performance improvements achieved by the proposed methodology in reducing phase lag.

### 4.2. Experiment Result and Analysis

#### 4.2.1. Computing Efficiency Analysis

**Figure 6:**
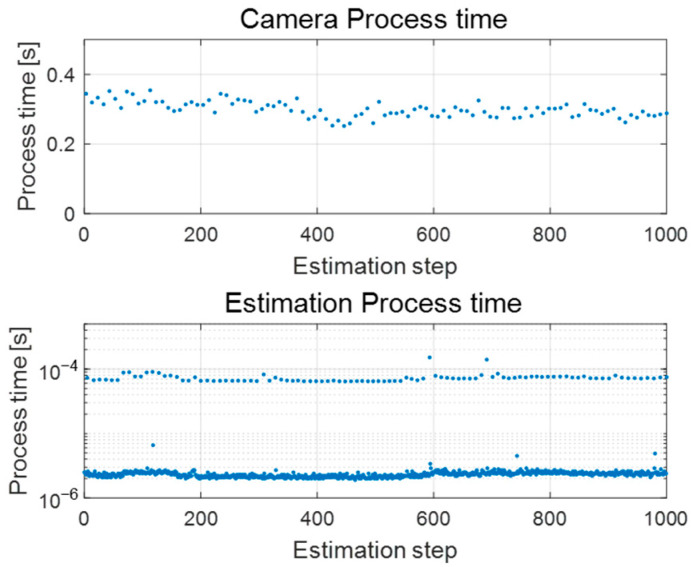
Camera and proposed estimation process time.

Before presenting the validation results, we first analyzed the processing time of the camera image-to-vehicle state extraction process and the proposed estimator. As mentioned earlier, despite the use of a pre-trained network for computational efficiency, the camera image processing required approximately 0.3 s per frame, as shown in Figure 6. This represents a considerable computational burden, even when executed on a high-performance laptop (AMD Ryzen 9 7845HX with NVIDIA RTX 4070). Such latency can critically impair the accuracy of vehicle state estimation. In contrast, the proposed estimator with signal delay compensation accurately estimates the current vehicle state regardless of the delay magnitude. Moreover, as illustrated in the second graph of Figure 6, the estimator itself operates at very high speed, ensuring real-time estimation without performance issues.

#### 4.2.2. Sine Wave Driving Scenario

**Figure 7:**
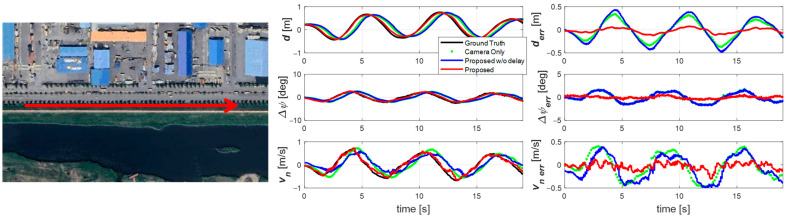
Experiment road and result for scenario 1. Sine wave driving on a straight road in the direction of the red arrow.

The first experiment was conducted at 60 km/h on a straight road during a one m-amplitude sine wave maneuver, as shown in Figure 7. The results (right plot of Figure 7) show that the raw camera measurement is significantly delayed. Without delay compensation, this delay propagates into the estimation results, as seen in the blue trace. However, with the proposed delay-compensated estimator, the results align closely with the ground truth and are free from phase lag. In addition, by accounting for signal characteristics, the estimator achieves a substantially lower error than the raw measurement.

**Figure 8:**
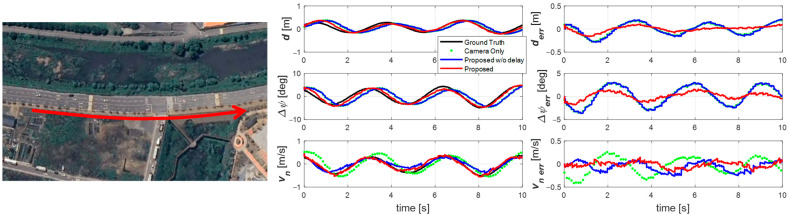
Experiment road and result for scenario 2. Sine wave driving on a curved road in the direction of the red arrow.

The second experiment (Figure 8) was performed at 30 km/h on a curved road with a 150 m radius. Although the maneuver was similar, the lower speed required larger and faster steering inputs, producing a larger Δ*ψ* while lateral offset and *v_n_* remained similar. Again, the proposed estimator accurately tracked the vehicle’s state, effectively compensating for delay even under large lateral motions at low speed. Due to the shorter time axis, it is particularly evident that the camera-only signals appear as sparse, delayed points. In contrast, the proposed estimator provided smooth, continuous estimates at 100 Hz without phase lag. The non-compensated estimator still lagged and failed to capture the vehicle’s motion fully.

#### 4.2.3. Double-Lane-Change Scenario

**Figure 9:**
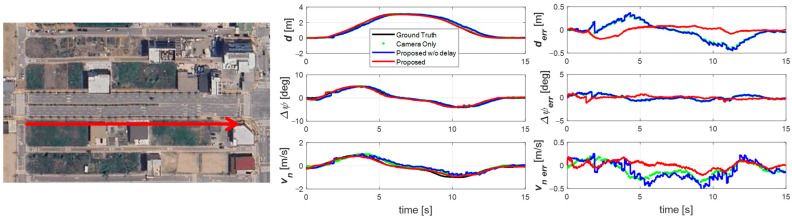
Experiment road and result for scenario 3. Double-Lane Change on a straight road in the direction of the red arrow.

**Figure 10:**
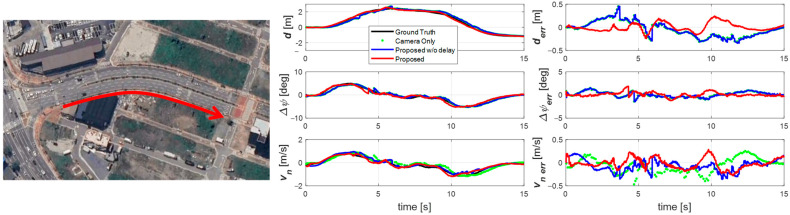
Experiment road and result for scenario 4. Double-Lane Change on a curved road in the direction of the red arrow.

The third and fourth scenarios (Figure 9 and Figure 10) involve Double-Lane-Change (DLC) maneuvers at approximately 40 km/h. Scenario 3 was conducted on a straight road, while scenario 4 was performed on a curved road with a 100 m radius.

In these cases, the lateral displacement was about 3 m (the width of a lane), resulting in lateral velocities roughly twice those of the sine wave experiments. The results again confirm that camera-only measurements and estimators without delay compensation suffer significant delays. In contrast, the proposed estimator closely tracks the ground truth without delay, even during large and rapid lateral movements.

#### 4.2.4. Overall Validation Analysis

For a comprehensive quantitative analysis of the four driving scenarios (sine wave maneuvers on a straight and a curved road, and double-lane changes), we calculated the Root Mean Square Error (RMSE) and Peak Error for each state variable. We summarized them in Table 3. The baseline was established using values directly measured from the camera and using the proposed estimator without delay compensation, and its performance was compared and analyzed against the proposed estimator.

The numerical results in Table 3 clearly demonstrate the superiority of the proposed methodology. The proposed estimator provides substantial quantitative improvements over the camera-only approach. In terms of RMSE, the proposed method reduced the error for *d* from a range of 0.121–0.201 m to 0.055–0.090 m, for Δ*ψ* from 0.486–2.040° to 0.269–0.821°, and for *v_n_* from 0.166–0.271 m/s to 0.068–0.117 m/s. Furthermore, the Peak Error also showed significant reductions for most states. The error for *d* decreased from 0.273–0.454 m to 0.122–0.233 m, and for *v_n_* from 0.357–0.475 m/s to 0.162–0.288 m/s. For Δ*ψ*, the Peak Error was generally reduced from 1.224–3.565° to 0.671–1.724°, with the exception of Scenario 3, where a slight increase from 1.522° to 1.811° was observed. Notably, the estimator that does not incorporate delay compensation shows performance that is either similar to or even worse than simply calculating the target state using only camera information. This is because the non-compensated estimator acts as a type of low-pass filter, causing the state to be estimated with a slight but persistent delay relative to the camera measurements.

This performance improvement can be attributed to two key elements of our research. First, the adaptive signal delay compensation algorithm effectively eliminates the substantial delay from camera image processing, allowing for accurate and real-time estimation of the vehicle’s current states. The significant error reduction, particularly in the high lateral velocity Double-Lane-Change (DLC) scenarios, proves the critical importance of delay compensation during dynamic maneuvers. Second, by fusing the vehicle dynamics model with IMU and steering angle sensor data, we were able to physically and logically estimate the intervals between the discontinuous and noisy camera measurements and effectively filter out noise by considering signal characteristics.

To validate the estimator’s performance while considering real-world driving scenarios, we selected sinusoidal driving and double-lane-change driving, which generate significant lateral motion on straight and curved roads, respectively, as validation scenarios. Since the proposed methodology consistently demonstrated performance across all these scenarios, we expect it to perform appropriately in most similar real-world driving situations. However, more complex driving conditions, such as rapid acceleration/deceleration or inclement weather, were not included in the scope of this study. Therefore, its performance in these typical driving environments requires further validation through future research.

In summary, the proposed estimator demonstrated robust and reliable performance across dynamic maneuvers and diverse road conditions. These results confirm that our methodology overcomes fundamental limitations of camera-based sensing, providing highly accurate real-time state information essential for ADAS and autonomous driving applications.

## 5. Conclusions

This paper presented a robust, real-time state estimator capable of determining a vehicle’s complete lateral state within the lane coordinate system. The primary contribution lies in the successful estimation of the lane-based lateral velocity—a critical yet unmeasurable state for motion prediction—using only a camera, IMU, and steering angle sensor, without reliance on high-cost sensors or HD maps. This was achieved by implementing a vehicle dynamics-based bicycle model within an Extended Kalman Filter (EKF), which proved superior to conventional kinematic approaches in capturing actual vehicle behavior, especially during dynamic maneuvers.

Furthermore, this dynamics-based framework inherently addresses the challenge of vision sensor latency. By predicting the vehicle’s state evolution during measurement delays, the proposed method effectively reduces estimation errors and phase lag. Validation with real driving data demonstrated that the estimator provides continuous and accurate state estimates across diverse scenarios, including lane changes and sine-wave maneuvers, confirming its reliability and real-time performance for practical applications.

Future work will focus on integrating the estimator with lateral controllers for closed-loop validation, testing robustness under adverse weather, and extending the approach to estimate the lane-coordinate-based states of surrounding vehicles through multi-sensor fusion.

## Figures and Tables

**Table 1:** Symbols.

	Description		Description
*d*	Lateral offset about the lane [m]	*m*	Vehicle mass [kg]
*ψ*	Heading angle in global [rad]	*I_z_*	Yaw moment of inertia [Nm]
*ψ_d_*	Heading angle of the lane [rad]	*a*	Distance from front axle to CG [m]
Δ*ψ*	Heading angle from the lane [rad]	*b*	Distance from rear axle to CG [m]
*u*	Longitudinal velocity [m/s]	*C_αf_*	Front tire cornering stiffness [N/rad]
*v*	Lateral velocity [m/s]	*C_αr_*	Rear tire cornering stiffness [N/rad]
*v* *_n_*	Lateral velocity about the lane [m/s]	*δ_f_*	Front Steer angle [rad]
*r*	Yaw rate [rad/s]	τ	Amount of signal delay [sec]
*r_d_*	Desired yaw rate along the lane [rad/s]	*z*	Measurements from the sensor

**Table 2:** Used vehicle parameter for the used model in the estimator.

Parameter	Value	Parameter	Value
*m*	1850 [kg]	*I_z_*	2800 [Nm]
*a*	1.35 [m]	*C_αf_*	86,000 [N/rad]
*b*	1.45 [m]	*C_αr_*	92,000 [N/rad]

**Table 3:** Root mean square error (RMSE) and Peak error (PE) of experiment results.

		Scenario 1			Scenario 2			Scenario 3			Scenario 4		
		Cam. ^1^	Est. ^2^	Prop. ^3^	Cam. ^1^	Est. ^2^	Prop. ^3^	Cam. ^1^	Est. ^2^	Prop. ^3^	Cam. ^1^	Est. ^2^	Prop. ^3^
*d* (m)	RMSE	0.201	0.256	0.055	0.121	0.131	0.061	0.171	0.177	0.090	0.183	0.192	0.077
	PE	0.369	0.490	0.122	0.273	0.288	0.160	0.454	0.454	0.233	0.418	0.450	0.196
Δ*ψ* (deg)	RMSE	1.003	1.008	0.269	2.040	2.059	0.821	0.577	0.559	0.481	0.486	0.511	0.332
	PE	1.803	1.773	0.671	3.565	3.626	1.724	1.522	1.442	1.811	1.224	1.242	1.150
*v_n_* (m/s)	RMSE	0.271	0.267	0.079	0.185	0.127	0.068	0.170	0.143	0.117	0.166	0.216	0.081
	PE	0.475	0.525	0.288	0.414	0.261	0.162	0.469	0.345	0.281	0.357	0.513	0.196

^1^ Camera only, ^2^ Estimator without delay compensation, ^3^ Proposed estimator with delay compensation.

## Data Availability

The data presented in this study are available on request from the corresponding author due to privacy.
